# Species limits, quarantine risk and the intrigue of a polyphagous invasive pest with highly restricted host relationships in its area of invasion

**DOI:** 10.1111/eva.12096

**Published:** 2013-08-21

**Authors:** Michelle A Rafter, James P Hereward, Gimme H Walter

**Affiliations:** School of Biological Sciences, The University of QueenslandBrisbane, Qld, Australia

**Keywords:** 28S, *Bryophyllum delagoense*, citrus, COI, cryptic species, gene flow, microsatellite, quarantine, *Scirtothrips aurantii*

## Abstract

*Scirtothrips aurantii* is a generalist horticultural pest in its native African range and recently established quite widely in Australia on the invasive succulent weed *Bryophyllum delagoense*. Paradoxically, this thrips is not polyphagous in its incursive range. The issue is principally one of quarantine. Will the thrips in Australia shift, perhaps adaptively, to citrus, and should the primary focus be on containment around Australian citrus, or does the real quarantine risk exist offshore with thrips present on citrus in Africa? We examined the phylogenetic relationships between *Bryophyllum*-associated thrips populations in Australia and populations sampled from various host plant species in South Africa (including *Bryophyllum*) using both CO1 and 28s markers. Eight variable microsatellite markers were developed to assess the extent of gene flow between the thrips on different hosts in South Africa. The COI phylogeny resolved *S. aurantii* into three distinct clades with samples collected from *B. delagoense* in South Africa and Australia representing a single clade, a second clade associated with *Gloriosa* lilies and the third with horticultural hosts. The microsatellite analysis confirmed that the populations associated with citrus and *Bryophyllum* do not hybridize with one another in sympatry. We conclude that the citrus-damaging thrips are not currently present in Australia and remain a serious quarantine concern in relation to Australian horticulture.

## Introduction

Effective quarantine is crucial to agriculture, trade and the environment. With respect to agriculture and trade, polyphagous species present extremely serious threats, especially if they have pest status (Boykin et al. 2012). Recent experience demonstrates, though, that not all individuals within generalist taxa achieve the full ecological amplitude attributed to that taxon [e.g. *Bemisia tabaci*, *Liriomyza trifolii* and *Copitarsia decolora* (Simmons and Scheffer 2004; Scheffer and Lewis 2006; Dinsdale et al. 2010)]. Further scrutiny has often shown that differences in host use, viral transmission, behaviour and so on can be associated with particular cryptic species later detected in the taxa involved (e.g. Scheffer and Lewis 2006; Malausa et al. 2007; Dinsdale et al. 2010). Understanding the source of such variation is crucial, not only to understanding the ecology and evolution of generalist taxa, but to understanding quarantine risk more completely. In this study, we examine the paradox of a pest species that is a putative generalist that has restricted host use in its invasive range. Our objectives are to (i) clarify the nature of the quarantine risk posed by the putative generalist and (ii) to assess the importance of an evolutionary approach to quarantine.

*Scirtothrips aurantii* Faure (Thysanoptera, Thripidae) is widely known as a generalist horticultural pest in its native African range, where it causes substantial economic loss to citrus producers (and other horticultural species) through the scarring their feeding induces on the surface of fruits (Samways et al. 1987; Lewis 1997; Grove et al. 1995). This species, known colloquially as the South African citrus thrips, was discovered in 2002 in Brisbane (Queensland, Australia), on *Bryophyllum delagoense* (Eckl. and Zeyh.) Schinz (Crassulaceae) (Anonymous 2003), and was soon acknowledged to be well established over a wide area (known to span Brisbane to Hervey Bay to Miles to Goondiwindi (approximately 946 400 km^2^) (M. A. Rafter, personal observation). *Bryophyllum delagoense* is itself an invasive succulent weed, mother-of-millions, that is under investigation for biological control in South Africa and Australia (Witt 2004; Witt and Rajaonarison 2004; Witt et al. 2006; Rafter et al. 2011; Palmer and Rafter 2012), but the incursion by *S. aurantii* into Australia was evidently accidental (Palmer 2005). Paradoxically, this generalist horticultural pest has not displayed the expected polyphagous tendencies in its incursive range. It is restricted in host use to plants in the family Crassulaceae in Australia (Anonymous 2003; Rafter et al. 2008; Rafter and Walter 2013a).

Several hypotheses were developed to explain the restricted host use of *S. aurantii* in Australia, including, (i) the invading population may have been so small as to have comprised only a small subset of the species' ecological potential (Anonymous 2003). That is, the number of founding individuals that entered Australia may have been so few that they carried only a small fraction of the total genetic variation in host use present in the source population. Even though the Australian population is now large, all individuals would consequently be genetically predisposed to feeding on *Bryophyllum* species. (ii) The *S. aurantii* population may, in Australia, be truly polyphagous and only temporarily restricted to *Bryophyllum*. If this alternative were correct, then *S. aurantii* individuals should move from *Bryophyllum* to its other recorded hosts, such as citrus and mango, with population increase (Morris and Mound 2004). Although the invasive population is now widespread and has been present for at least 11 years (approximately 100 generations), these thrips may conceivably still move to horticultural hosts, although we know of no published records of a serious pest species that has shown such a delayed response in this regard. (iii) The species name *S. aurantii* may mistakenly include more than one cryptic species, a possibility that is often overlooked when exploring generalist host relationships (Paterson 1991; Walter 2003). Such circumstances can be subtle because genetically independent species may show no morphological differences from one another, a feature not uncommon in small insects (Coetzee 1989; Fernando and Walter 2008; Condon et al. 2008).

When restricted to a single host species growing in the laboratory, *S. aurantii* collected from *B. delagoense* in Australia will feed, with some delay, on macadamia (Macadamia integrifolia) and mango (Mangifera indica). In contrast, it readily feeds on the crassulaceous ornamental Kalanchoe blossfeldianna (Rafter et al. 2008; Rafter and Walter 2013a). However, when presented with two or more species simultaneously (one of them *B. delagoense*), they always colonize *B. delagoense* rather than the alternative species presented (Rafter and Walter 2013a). Further, field surveys in the area of *S. aurantii* incursion within Australia demonstrated conclusively that under natural conditions, *S. aurantii* in Australia is consistently present on *B. delagoense* (Rafter et al. 2008; Rafter and Walter 2013a). This contrasts strongly with the polyphagous habits of this species in its native range in Africa.

Analyses of mate recognition between individual thrips of the Australian *Bryophyllum* population and a South African horticultural population, using reciprocal cross-mating tests, indicate that mating between the two host-associated populations occurs at a much lower frequency than mating within each of the two populations, even in confinement (Rafter and Walter 2013b). This suggests the presence of host-associated cryptic species. Different closely related species may still cross-mate in laboratory confinement, probably because any long distance cues that differentiate them in nature are circumvented (Fernando and Walter 2008), and many herbivorous insects have mating behaviour that is closely associated with their host plant (Claridge et al. 1985, 1988; Drosopoulos 1985).

The observations summarized above are difficult to reconcile with molecular data previously published on this putative generalist species (e.g. Morris and Mound 2004; Hoddle et al. 2008). An analysis of a 487-bp fragment of the mitochondrial DNA (mtDNA) COI gene and a 614-bp fragment of the internal transcribed spacer two region (ITS2) amplified from three Australian individuals and 24 thrips from South Africa revealed two divergent lineages with up to 3% pairwise differences (Morris and Mound 2004). All three Australian individuals clustered within one of these clades, but South African samples collected from citrus plants were present in both of these two clades (Morris and Mound 2004). The *Bryophyllum-*associated insects in Australia and South Africa were therefore said to be of the same species as populations on citrus in South Africa, but the population in Australia may represent a ‘strain’ that may still expand its host preferences.

In an investigation of taxonomic relationships among *Scirtothrips* species, including *S. aurantii*, Hoddle et al. (2008) analysed a 663-bp fragment of mtDNA COI gene and the 28S-D2 domain of the large-subunit rRNA. The analysis included one *S. aurantii* individual collected from *B. delagoense* in Australia and three thrips collected from citrus in South Africa. Thrips from both countries formed a monophyletic clade with 100% bootstrap support in this analysis, although the two sequences deposited to GenBank (citrus, South Africa: EU100994 and *Bryophyllum*, Australia: EU100995) have a 3.3% pairwise nucleotide difference. These results were seen to support the earlier conclusion that the invasive thrips on *Bryophyllum* in Australia are probably of the same species as the polyphagous populations in South Africa (Hoddle et al. 2008).

Resolution of the species status of the host-associated populations of *S. aurantii* is imperative as the potential quarantine risks to Australian horticulture remain unclear. Is the *Bryophyllum* population present in Australia a potential risk to horticulture? Is the population likely to shift (perhaps adaptively) onto citrus, or does the risk lie in our misclassification of a host-specific cryptic species? Further, a full resolution of the genetical status of the various host-associated *S. aurantii* populations will also help guide management strategies for pest populations in South Africa.

## Materials and methods

### Sampling and DNA extraction

Surveys of known host plants of *S. aurantii* were conducted in South Africa over a 2-week period during February 2010 in several sites across three locations: Pretoria (25°44′S 28°11′E), Nelspruit (25°27′S 30°59′E) and Wellington (33°38′S 18°59′E) (Rafter and Walter 2012). Eight plant species were sampled (Table 1). Samples from Australian *B. delagoense* were taken in March 2010 across two locations, Brisbane (27°28′S 153°01′E) and Miles (26°40′S 150°11′E) (Table 1). All plants were sampled by beating foliage over a white tray. Dislodged thrips were placed into 2-mL glass vials containing 95% ethanol, using a fine brush. Subsamples of 5–15 thrips were placed into 2-mL glass vials containing AGA [Alcohol (60%), glycerine, acetic acid (10:1:1)] for slide mounting (see Mound and Marullo 1996) and morphological identification (Mound and Palmer 1981; Moritz et al. 2004; Mound and Stiller 2011). Details of species identifications of thrips contained in the subsamples are presented by Rafter and Walter (2012). Genomic DNA was extracted with 10% Chelex (Walsh et al. 1991). Individual Chelex samples containing the exoskeleton of extracted thrips have been deposited in a −20°C molecular specimen freezer at the University of Queensland and are available for morphological examination upon request.

**Table 1:** Collection information for samples used in this study

Code	Location	*N*	Coordinates	Sampling date	Plant host (Family)
MBd	Miles	39	26°40′S 150°11′E	19 March 2010	*Bryophyllum delagoense* (Crassulaceae)
BBd	Brisbane	25	27°28′S 153°01′E	24 March 2010	*Bryophyllum delagoense* (Crassulaceae)
NBd	Nelspruit	46	25°27′S 30°59′E	4–5 February 2010	*Bryophyllum delagoense* (Crassulaceae)
NCs	Nelspruit	21	25°27′S 30°59′E	5 February 2010	*Citrus sinensis* (Rutaceae)
NMi	Nelspruit	21	25°27′S 30°59′E	5 February 2010	*Macadamia integrifolia* (Proteaceae)
NCp	Nelspruit	25	25°27′S 30°59′E	4 February 2010	*Caesalpinia pulcherrima* (Fabaceae)
PCs	Pretoria	23	25°44′S 28°11′E	3 February 2010	*Citrus sinensis* (Rutaceae)
PGs	Pretoria	32	25°44′S 28°11′E	3 February 2010	*Gloriosa superba* (Colchinaceae)
PCm	Pretoria	21	25°44′S 28°11′E	3 February 2010	*Crassula multicava* (Crassulaceae)
PKb	Pretoria	12	25°44′S 28°11′E	3 February 2010	*Kalanchoe blossfeldiana* (Crassulaceae)
WCs	Wellington	29	33°38′S 18°59′E	10 February 2010	*Citrus sinensis* (Rutaceae)
WPg	Wellington	28	33°38′S 18°59′E	10 February 2010	*Punica granatum* (Lythraceae)

*N* = number of individuals screened and included in the microsatellite analysis.

### COI/28S sequencing and phylogenetic analysis

To replicate the test of Morris and Mound (2004), we amplified a similar region (571 bp) of the COI gene using primers C1-J-1718 and C1-N-2329 (Simon et al. 1994). This primer combination failed to amplify the desired fragment in the *S. aurantii* individuals collected from *Gloriosa superba*, so with these individuals, a 430-bp fragment (internal to the 571-bp fragment mentioned above) from these individuals was amplified using primer C1-J-1718 in conjunction with HCO2198 (Folmer et al. 1997). PCR was performed with Platinum *Taq*™ (Invitrogen, Carlsbad, CA, USA), 0.2 μm of each primer and 4 mm of MgCl_2_. PCR cycling conditions consisted of 95°C for 10 min followed by an increment of one degree per cycle (nine cycles) from 45 to 54°C annealing temperature (60 s) and 25 additional cycles at 54°C. Denaturation was 95°C for 30 s, and elongation was 72°C for 45 s.

A 715-bp fragment of the D2–D3 region of 28S was amplified with the primers S3660 (28SF, Dowton and Austin 1998) and A335 (28Sb, Whiting et al. 1997). PCR was performed using My *Taq*™ (Bioline, Taunton, MA, USA), 0.2 μm of each primer and the standard buffer (including 1.5 mm MgCl_2_). PCR cycling conditions consisted of 95°C for 10 min followed by 35 cycles at 52°C (45 s). Denaturation was 95°C for 30 s, and elongation was 72°C for 45 s. Amplicons for both COI and 28S samples were sequenced bidirectionally on an ABI 3730 (Macrogen Inc., Seoul, South Korea). Sequences were edited using CodonCode Aligner, aligned using Geneious™ (Drummond et al. 2010), and the suitability of 211 models of molecular evolution was assessed with jModelTest (Posada 2008). Outgroups were obtained from the nucleotide collection (*nr*, GenBank), and Bayesian trees constructed on the final 390-bp alignment using the most likely model (GTR+I+G) with MrBayes (Huelsenbeck and Ronquist 2003) run with eight chains for 1 000 000 iterations. All sequences included in the phylogenetic analyses have been deposited in GenBank COI accession numbers = KF287433–KF287514 and 28S = KF287515–KF287635.

### Microsatellite primer development

**Figure 1:**
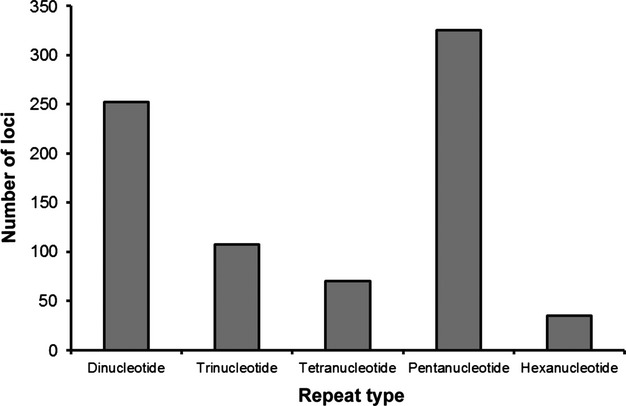
Frequency of repeat classes for *Scirtothrips aurantii* microsatellites longer than eight repeats (excluding mononucleotides).

Genomic DNA was extracted from a pooled sample of 500 individuals collected from *B. delagoense* in Brisbane, Australia, using DNeasy kits (Qiagen Inc., Hilden, Germany) and sequenced as 1/16 of a GSFLX 454 (Roche Diagnostics Corp., Branford, CT, USA) plate by the Australian Genome Research Facility (Brisbane, Qld, Australia). This provided >38 000 raw reads of between 30 and 630 bp in length. Microsatellites with a minimum of eight repeats were identified from these sequences using the program MSATCOMMANDER (Faircloth 2005) so that the number of loci obtained for each repeat type could be plotted (Fig. 1). Pentanucleotides were the most abundant repeat type in the *S. aurantii* sample (Fig. 1).

We used the QDD 2.1 algorithm (Meglécz et al. 2010) to remove sequences below 80 bp, make consensus sequences (above 95% sequence identity), identify reads containing microsatellites and discard reads that may be multiple copy within the genome (transposable elements). We then designed primers within QDD for amplicons between 90 and 400 bp, optimal Tm of 60°C and default values for the remainder of the settings using Primer3 (Rozen and Skaletsky 2000). Twenty-four primer pairs were initially screened against eight *S. aurantii* (four from a *B. delagoense* population in Australia (M), two from *B. delagoense* in South Africa (NBd) and two from citrus in South Africa (NC) (Table 1)). These 24 loci were screened for amplification efficiency, dye performance and polymorphism by capillary electrophoresis on an ABI3730 (Macrogen Inc.). Subsequent optimization was conducted on 45 males to confirm their haploidy and to screen the loci further for nonspecific amplification. Subsequent analysis of gene frequencies was restricted to the diploid females. Further primer optimization was conducted on individuals from three host-associated populations of thrips [M, NBd and NC (Table 1)]. Twenty-one loci were tested on these individuals for consistent amplification success and the presence of null alleles. Five loci failed to amplify in the citrus population, six amplified multiple fragments, two were monomorphic, and eight yielded polymorphic loci (for locus details, see Table 2 and Table S1). We provide primer options for an additional 1001 microsatellites identified by the QDD program as supplementary data (Table S3). These primer options are all given a quality score as described in the QDD documentation.

**Table 2:** Characteristics of the eight *Scirtothrips aurantii* microsatellite loci used in this study, locus name, the repeat sequence targeted, fluorescent dye used, size range, number of alleles (Na), mean allelic richness, Hardy–Weinberg deviations (HW), mean estimated null allele frequency (Null) and locus-specific *F*_ST_ [with exclusion of null alleles (ENA) correction for null alleles]. Global *F*_ST_ across all markers and populations = 0.28. Sampled populations were split into three groupings for calculations of NA and allelic richness, with ‘Aust. Bryo.’ = Australian *Bryophyllum* (M and BBd) (with specific population codes as detailed in Table 1), ‘SA Bryo.’ = South African *Bryophyllum* (NBd and PK) and ‘SA Hort.’ = horticultural hosts in South Africa (NC, NM, NP, PC, PCm, WC and Wpom). For population specific values, refer Table S2

				Na			Allelic richness						
Locus	Repeat motif	Dye	Size range	Aus. Bryo.	SA Bryo.	SA Hort.	Aus Bryo.	SA Bryo.	SA Hort.	HW*	Null	Locus-specific *F*_ST_	GenBank accession numbers
SACT02	CCGGG	PET	176–248	1	10	13	1.00	3.09	1.18	1	0.05	0.38	KF287431
SACT05	CCCG	FAM	223–281	7	9	19	4.34	4.71	1.89	4	0.17	0.14	KF287432
SACT06	CT	VIC	142–208	2	8	16	2.00	4.78	1.62	1	0.04	0.30	KF287427
SACT13	AGGCC	PET	210–297	2	9	15	1.87	5.14	1.85	1	0.04	0.45	KF287426
SACT17	ACGGG	NED	170–232	4	9	17	3.29	3.79	1.88	1	0.01	0.17	KF287428
SACT18	GGGCT	FAM	120–172	4	5	11	3.55	4.40	1.35	2	0.07	0.39	KF287429
SACT19	CCGGG	NED	113–165	3	8	11	2.87	4.45	1.79	2	0.09	0.22	KF287430
SACT52	AGT	PET	238–340	4	6	24	2.57	4.71	1.93	6	0.04	0.15	KF287425

* Number of significant deviations from HWE out of 11 populations after Bonferroni correction for multiple tests.

### Microsatellite genotyping

The eight polymorphic loci were amplified across 290 *S. aurantii* individuals sampled from 11 geographical host-associated populations (Table 1). Fluorescent dye was added to the fragment by the addition of M13 tails (Schuelke 2000) (Table 2). Loci were amplified in 12 μL reaction mixtures containing 0.03 units of My *Taq*™ (Bioline), 1× buffer, 0.1 μm of forward primer, 0.2 μm fluorescently labelled M13 tails and 0.2 μm of reverse primer. Amplification conditions were as follows: 10-min initial denaturation (95°C); a two-step cycle with 25 cycles of 95°C for 25 s, annealing of 57°C for 30 s and 72°C for 45 s and 10 cycles of 95°C for 30 s, annealing of 54°C for 30 s and 72°C for 45 s; and final extension of 72°C for 10 min. PCR product was separated (one locus per dye) by capillary electrophoresis on an ABI3730 (Macrogen Inc.). Microsatellite peaks were confirmed and binned manually using the program GeneMarker®, v. 2.2.0 (Softgenetics, State College, PA, USA).

### Genetic diversity and Hardy–Weinberg equilibrium

The number of alleles (Na) per locus was calculated in GENALEX 6 (Peakall and Smouse 2006), and allelic richness was calculated in FSTAT (Goudet 1994). Conformity to Hardy–Weinberg equilibrium was tested using the exact tests implemented in Genepop, v. 4.1 (Rousset 2008). Null allele frequencies (null) were estimated using the expectation maximization algorithm of Dempster et al. (1977) as implemented in FreeNA (Chapuis and Estoup 2007). Locus-specific global *F*_ST_ values (Weir 1996) were computed with the exclusion of null alleles (ENA) algorithm implemented in FreeNA (Chapuis and Estoup 2007). We tested for genetic evidence of a recent population bottleneck event associated with the establishment of *S. aurantii* on *Bryophyllum* in Australia using the sign test, Wilcoxon test and mode-shift analysis implemented in the program BOTTLENECK, v.1.2.02 (Cornuet and Luikart 1996), under all three mutational models.

### Genetic differentiation and gene flow across host plant-associated populations

To assess genetic differentiation between populations of *S. aurantii*, pairwise *F*_ST_s were calculated with the ENA algorithm in FreeNA (Chapuis and Estoup 2007), and exact tests of genotypic differentiation were performed in Genepop (Rousset 2008). These population-based analyses of differentiation require that populations have been defined correctly *a priori*. We therefore used the individual-based clustering algorithm implemented in STRUCTURE (Pritchard et al. 2000) to assign individuals to various specified numbers of clusters (K) within a Markov chain Monte Carlo framework, using gene frequencies. We used both the ‘admixture’ and ‘no-admixture’ models. In the former, individuals are allowed shared ancestry between populations, whereas the ‘no-admixture’ model assumes that populations are discrete. These models were run with all eight loci and with locus SACT05 (which showed evidence of null alleles in some populations) removed. The results were the same regardless of the inclusion of this locus, so all subsequent analyses were performed on all eight loci. A burn-in of 50 000 iterations was used with a further 500 000 iterations and did not allow the use of population designations for the inference of cluster membership. The algorithm was run using the admixture model initially, with 20 replicates of each designated value of *K* (*K* = 1 to K = 10). The most likely value of *K*, given the data, was inferred by the Evanno et al. (b117) method implemented in STRUCTUREHARVESTER (Earl and vonHoldt 2011). Twenty replicates were performed for *K* = 2 and *K* = 3 under both the ‘admixture’ and ‘no-admixture’ models; these results were then permuted and averaged using CLUMPP (Jakobsson and Rosenberg 2007) and plotted using ‘distruct’ (Rosenberg 2004).

## Results

### COI and 28S phylogenetic analyses

**Figure 2:**
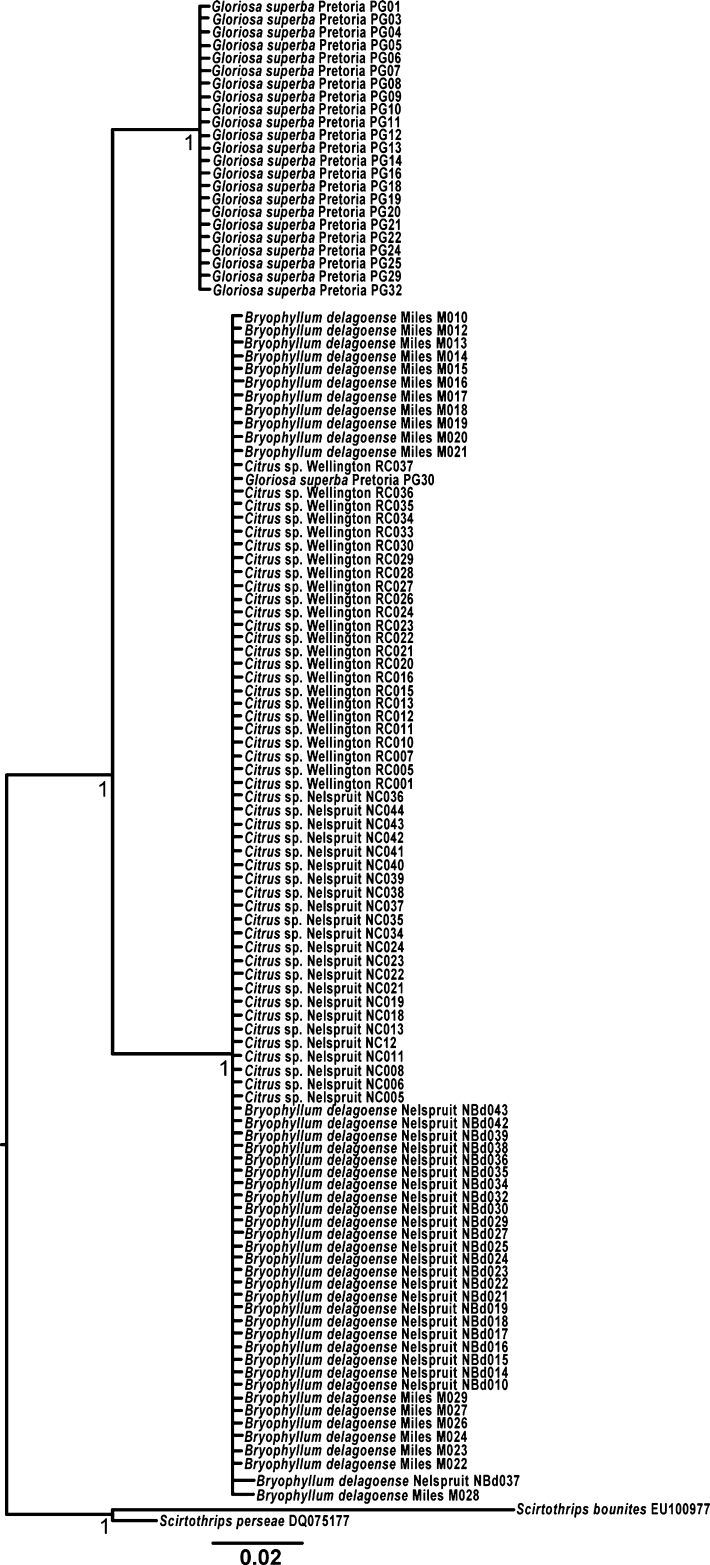
Bayesian consensus tree computed with MrBayes from a 390-bp fragment of the D2–D3 region nuclear large-subunit ribosomal RNA gene (28S), with *Scirtothrips perseae* and *Scirtothrips bounites* as the rooted outgroups (GenBank accession numbers DQ075178 and EU100977, respectively). The collection locality for each individual follows the name of the host plant from which it was collected. All localities are South African except for Miles (Queensland, Australia).

The 28S phylogeny resolved two monophyletic clades, each with 100% bootstrap support within the species currently defined as *S. aurantii* (Fig. 2). Thrips associated with *Gloriosa superba* (except for PG30, see Discussion) formed a single clade with a mean pairwise nucleotide difference of 3.3% from the clade associated with *B. delagoense* (from both Australia and South Africa) and citrus (Fig. 2). This slowly evolving locus indicated no divergence between populations sampled from citrus and *B. delagoense*.

**Figure 3:**
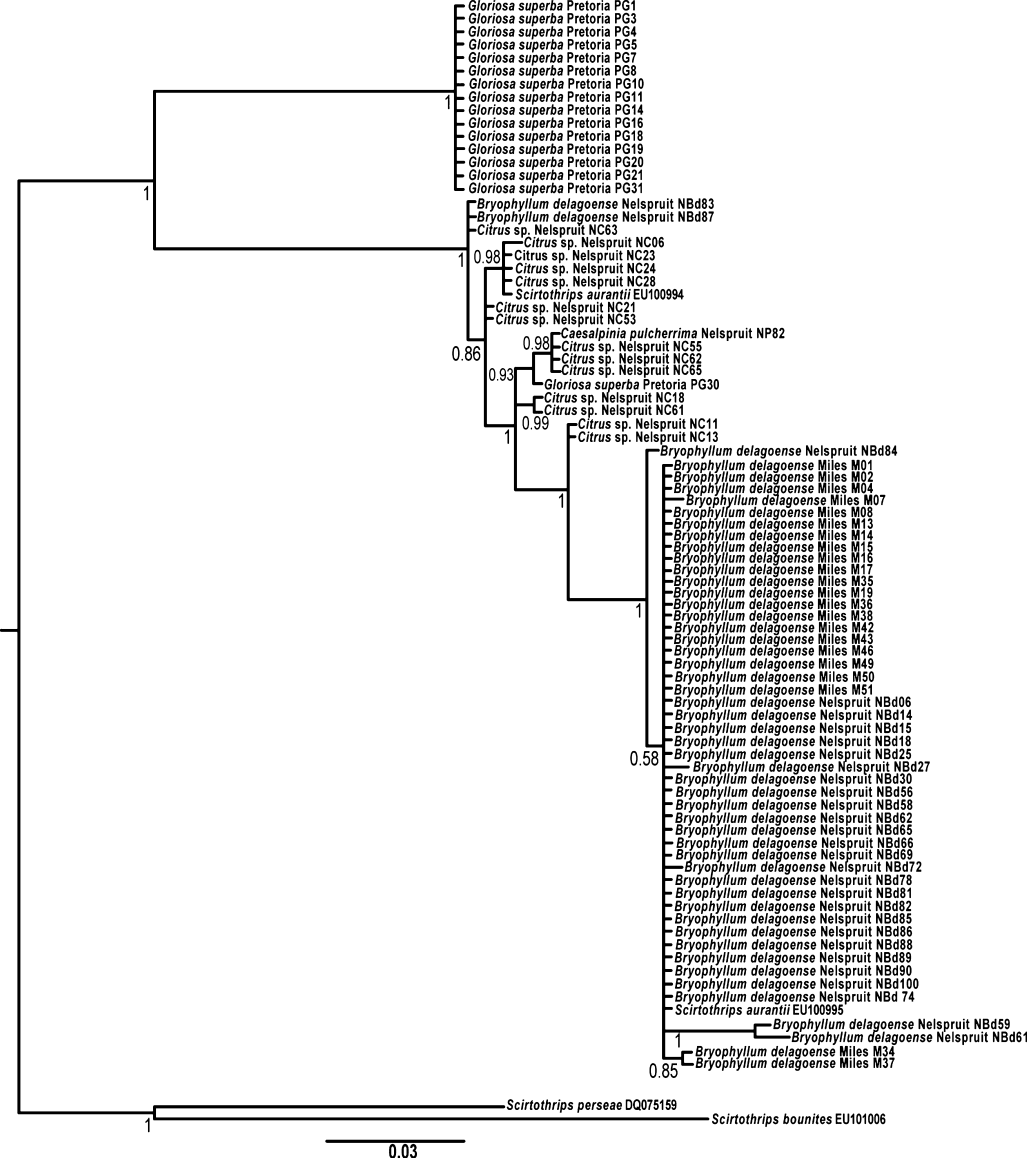
Bayesian consensus tree computed from 430-bp sequences of mtDNA COI gene, with *Scirtothrips perseae* and *Scirtothrips bounites* as the rooted outgroups (GenBank accession numbers DQ75158 and EU101006, respectively). Sequences of *Scirtothrips aurantii* individuals from previous studies (GenBank accession numbers EU100994 and EU100995) are also included in the tree. Host plants and localities as for Figure 2.

The more rapidly evolving COI fragment revealed divergent lineages associated with citrus and *B. delagoense* (Fig. 3). Samples collected from *B. delagoense* in South Africa and Australia form a single monophyletic clade (with the exception of individuals NBd83 and NBd87, see Discussion), with 99% bootstrap support. Thrips associated with *G. superba* again form a single clade with 100% bootstrap support (Fig. 3). The average pairwise difference between the citrus clade and the *B. delagoense* clade was 2.96% (Fig. 3), and the mean pairwise difference between the *G. superba* clade and the citrus and *B. delagoense* clades was 12.3% and 13.5%, respectively (Fig. 3).

### Genetic diversity and Hardy–Weinberg equilibrium

The eight microsatellite markers developed in this study that did cross-amplify across the host-associated populations were polymorphic in both the Australian and South African samples (Table 2 and Table S2). Polymorphism was lower in the Australian samples (mean alleles per locus = 3.37) than the South African ones (mean alleles per locus for *Bryophyllum* = 8.00 and the horticultural hosts combined = 15.75). Under all three mutational models (stepwise, infinite alleles and two-phase), the sign test and Wilcoxon tests of heterozygote excess were not significant (at *P *= 0.05). The mode-shift analyses of the two Australian *Bryophyllum* populations also returned normal L-shaped distributions. There is thus no evidence for a recent genetic bottleneck event in any of the populations sampled based on the microsatellite data. Low levels of null alleles were inferred for all loci, but SACT05 had a relatively high null allele frequency (17%, Table 2). The structure analyses were run with and without this locus, and the results were identical.

### Microsatellite analysis of gene flow across host plants

Global *F*_ST_ was high (0.28), and from the pairwise values (Table 3), it is evident that this is due to the *Bryophyllum*/citrus comparisons. Indeed, *F*_ST_ estimates range from 0.03 to 0.17 within citrus populations, while they range from 0.22 to 0.53 between populations collected from different hosts (*Bryophyllum* versus citrus). In sympatry, these pairwise comparisons between crassulaceous hosts and citrus hosts were also high (Nelspruit = 0.22, Pretoria = 0.36).

**Table 3:** Pairwise estimates of *F*_ST_ inferred using ENA correction and exact tests of genotypic differentiation (as indicated by superscripts defined in the footnote below), between the 11 sampled populations (codes in Table 1) in which the eight microsatellite loci listed in Table 2 could be amplified (see text)

Population	NBd	NCs	NMi	NCp	MBd	BBd	PCs	PCm	PKb	WCs
NCs	0.225*									
NMi	0.186*	0.015**								
NCp	0.244*	0.023*	0.002**							
MBd	0.150*	0.417*	0.375*	0.413*						
BBd	0.217*	0.444*	0.383*	0.436*	0.139*					
PCs	0.251*	0.028*	0.005**	−0.002**	0.418*	0.454*				
PCm	0.260*	0.060*	0.038*	0.020*	0.440*	0.469*	0.030*			
PKb	0.258*	0.339*	0.316*	0.346*	0.456*	0.513*	0.362*	0.374*		
WCs	0.334*	0.167*	0.145*	0.140*	0.500*	0.528*	0.152*	0.156*	0.439*	
WPg	0.326*	0.161*	0.156*	0.152*	0.500*	0.528*	0.161*	0.171*	0.419*	0.017**

*P*-values from exact tests of genotypic differentiation [significance level Bonferroni corrected for multiple comparisons (0.05/55) = 0.0009]: Not significant

‘**’ =>0.0009

‘**’ =<0.0009.

**Figure 4:**
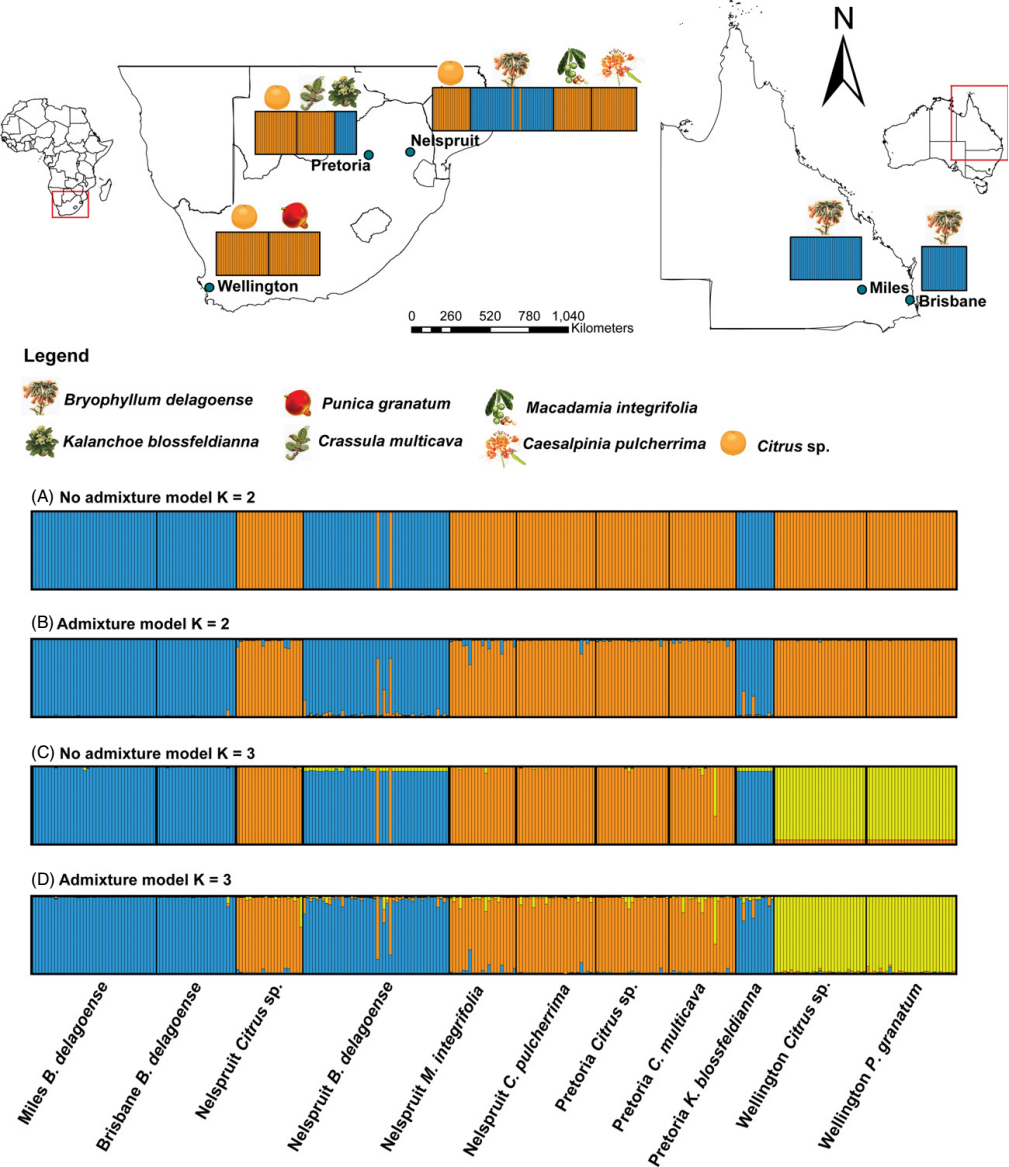
Below: STRUCTURE outputs (with locality and host plant species listed across the bottom of the diagram) for (A) ‘no-admixture’ model *K* = 2, (B) ‘admixture’ model *K* = 2, (C) ‘no-admixture’ model *K* = 3 and (D) ‘admixture’ model *K* = 3. Bars within the STRUCTURE plots represent individual thrips, and the colours indicate the posterior probability of assignment of that individual to a particular cluster. Top: STRUCTURE output (‘no-admixture’ model, *K* = 2) plotted on maps of South Africa and Australia by sampling locality and host plant.

The most likely value of *K* based on 20 replicates (*K* = 1 to *K* = 10) was *K* = 2, with a second most likely value at *K* = 3 (Fig. S1), using the Evanno et al. (b117) delta K method. The ‘no-admixture’ model presented in Fig. 4 is the most appropriate given the results of the COI phylogenetic analysis (Fig. 3), in which discrete monophyletic clades correspond to particular host plants. With *K* = 2 and the ‘no-admixture’ model, the thrips collected from *B. delagoense* and *K. blossfeldianna* were assigned with practically 100% posterior probability to a single cluster (blue bars, Fig. 4). Only two individuals were exceptional in this regard, namely NBd 83 and 87 (Fig. 4). These are dealt with further in the discussion. Those insects sampled from citrus, *M. integrifolia, C. multicava, P. granatum* and *C. pulcherrima*, were all assigned to the second cluster (orange bars, Fig. 4).

When the structure algorithm was run with *K* = 3, the Wellington citrus and *P. granatum* thrips populations were assigned to a single cluster (yellow bars), separate from citrus populations sampled further north in South Africa (orange bars) (Fig. 4C,D). Populations sampled from *B. delagoense* in Australia were assigned to the same cluster (even with *K* = 3) as populations sampled from *K. blossfeldianna* and *B. delagoense* in South Africa despite at least 10 years of geographical isolation (Fig. 4).

## Discussion

The genetic divergence (Fig. 3) between *Bryophyllum*-associated *S. aurantii* and those associated with citrus, together with the lack of gene flow across these host-associated populations (Fig. 4), demonstrates that the thrips currently classified as *S. aurantii* do not belong to a single species but rather comprise a suite of at least three cryptic species, one on *Gloriosa superba*, one on *Bryophyllum delagoense* and *Kalanchoe blossfeldianna* (and possibly other crassulaceous species) and the third on *Citrus* sp., *Macadamia integrifolia, Caesalpinia pulcherrima, Punica granatum* and *Crassula multicava*. The insects on *Bryophyllum* in Australia clearly originated from *Bryophyllum* in South Africa are confined to that host plant and some other Crassulaceae in that country, and they are therefore not likely to move onto citrus from *Bryophyllum* in Australia. This conclusion is explained and justified below, and the implications for quarantine are discussed.

The average pairwise difference between the citrus clade and the *B. delagoense* clade is 2.96% for COI gene (Fig. 3). These host-associated lineages are thus likely to have diverged around 1mya based on the generally accepted rate of COI divergence in insects of 2% per million years (DeSalle et al. 1987). The thrips associated with *G. superba* (except for individual PG30) formed a single clade distinct from the other *S. aurantii* populations sampled with respect to both the 28S and COI markers (Figs 2 and 3). There was a 3.3% nucleotide difference between the *G. superba* clade and the single clade associated with *B. delagoense* and citrus for the 28S marker and 12.3% and 13.5% from the citrus and *B. delagoense* clades, respectively, for the COI marker. The thrips associated with *G. superba* are likely to have split from the *B. delagoense*/citrus complex of thrips about 4-8mya (DeSalle et al. 1987). Thrips sampled from *G. superba* are so different genetically that none of the microsatellites designed specifically for this study of *S. aurantii* would amplify for this population.

Clearly, no gene flow was detected between thrips sampled from *B. delagoense* and those sampled from citrus, *M. integrifolia* and *C. pulcherrima* in Nelspruit (an area of sympatry, under 5 km) (Fig. 4). This lack of gene flow is also evident in the other area of sympatry that was sampled, Pretoria, for no gene flow was evident between populations sampled from *K. blossfeldianna* (another crassulaceous host) and populations sampled from *C. multicava* and citrus (Fig. 4). The two molecular approaches used in this study (microsatellite analysis of gene flow and COI-based phylogeny) both indicate a lack of gene flow between the thrips on *Bryophyllum* (and other crassulaceous hosts) and populations associated with horticulture, even in areas of sympatry. Furthermore, the microsatellite markers developed using the Australian *Bryophyllum* population also worked well for the South African *Bryophyllum* population, but not all of these markers could be amplified in South African horticultural populations. Five of the 21 markers screened during primer development were thus rejected, and their failure to cross-amplify is likely a consequence of sequence divergence between these two lineages in the primer binding sites.

Our analyses confirm that these host-associated populations of *S. aurantii* (*sensu lato*) do not hybridize under natural conditions. However, thrips from *Bryophyllum* will cross-mate with thrips from citrus and other horticultural hosts in confinement under laboratory conditions, although the frequency of mating between them is significantly lower than in control crosses of thrips collected from the same host plant species (Rafter and Walter 2013b). Many species that are discrete in nature will, nevertheless, mate in confinement (and even produce viable offspring) (Claridge et al. 1985, 1988; De Winter 1995; Sun et al. 2011; Li et al. 2012). This may be because their specialist host plant associations preclude the sexes from meeting or that long distance cues (e.g. pheromones) are crucial to bringing potential mates together in nature (Fernando and Walter 2008). The *S. aurantii* from *Bryophyllum* comprise a host-associated gene pool that is independent of the ‘*S. aurantii*’ associated with citrus and other horticultural hosts. These host-associated populations are therefore separate species, and they are referred to as such below.

When the structure analysis was performed under the more relaxed ‘admixture’ model, and with *K* set to three, not all individuals were resolved into separate clusters with 100% posterior probability (Fig. 4D); however, it is possible this is a consequence of the difficulty in cross-amplifying these markers and the resultant alleles that failed to amplify in some populations (null alleles) (Table 2). We have tested the reproductive mode of these thrips and clearly established that these two host-associated species are both haplodiploid. Null alleles can arise from inbreeding, and it is possible for thrips females to produce male progeny asexually and then mate with them, and in small colonies, this can lead to an observed decrease in heterozygosity (Yang et al. 2012). Such a possibility would, however, have to be tested specifically for thrips in the *S. aurantii* species complex.

The analyses of the results presented in this article clearly support the view that the name *S. aurantii* covers a complex of three species, and perhaps even more. The results also allow comment on the two other explanations of the host plant associations of ‘*S. aurantii*’ in Australia. The lower number of alleles and decreased allelic richness evident in the Australian *Bryophyllum* thrips compared with populations of South African *Bryophyllum* thrips indicates that a relatively small number of individuals established in Australia, so it is likely to have been a single event (Table 2). However, the results provide no evidence for the occurrence of a recent genetic bottleneck in the Australian population, so the host plant restriction of the thrips in Australia is not the result of only a small fraction of the total genetic variation in host preference entering Australia as previously hypothesized (Anonymous 2003). As to the question of whether the ‘*S. aurantii*’ present on *Bryophyllum* in Australia will move onto citrus and other horticultural hosts, the evidence available from South Africa suggests this is not at all likely. The population present in Australia belongs to a cryptic species that has associations with crassulaceous hosts, does not exchange genes with the *S. aurantii* species present on horticultural hosts and has been an independent lineage for an estimated 1 million years.

Two individual thrips associated with *B. delagoense* in Nelspruit (NBd 83 and 87) and one associated with *G. superba* in Pretoria (PG30) were assigned to the ‘citrus’ clade rather than that formed by the other individuals from their respective host plants. Three possible explanations for their assignment to the citrus clade are possible. (i) They may have been incorrectly sampled as these highly mobile insects are <1 mm in length and could have flown into the collecting tray, or they may have been present on collecting equipment from previous samples despite decontamination efforts. (ii) Individuals possibly do fly between these plants at a low rate. (iii) They do fly and feed on alternative plants at a low rate but they do not mate on these alternatives [as evidenced by the lack of gene flow between the host-associated populations (Fig. 4)]. In other words, these three individuals (of 290 included in the analysis) could have been miss-assigned or they are incidental on *Bryophyllum* and *G. superba* at very low frequencies. These possible explanations could be resolved by amplifying and sequencing any ingested chloroplast markers to determine exactly which host plants these specific individuals had recently fed upon (Hereward and Walter 2012) and by further collection and genetic analysis.

Previous genetic screening of 25 thrips individuals collected from citrus in South Africa and *Bryophyllum* in both South Africa and Australia also assigned some individuals to lineages different to the rest of the individuals collected off a host plant (Morris and Mound 2004). Also, several individuals had a nuclear sequence typical of the lineage associated with one of the hosts and the mitochondrial sequence of the alternative lineage. Two individuals were even associated with both of the highly divergent nuclear sequences (Morris and Mound 2004). None of these discrepancies were ever detected between the mitochondrial data and nuclear markers (microsatellites) in our study. Given that the DNA extraction method of Morris and Mound (2004) yielded very weak genomic DNA, the unusual assignment they report may well have resulted from contamination during PCR amplification.

### Correct species delimitation and quarantine risk

If cryptic species within the *S. aurantii* taxon had remained undetected, the priority in Australia would be a continued focus on the incursive population and keeping it away from horticulture. This focus, supported by incorrect species delimitation, carries the potential danger of removing attention from the quarantine risk still posed to Australia by the citrus-associated population in South Africa. Given the data and analysis presented here, the Australian containment emphasis should be shifted from the Australian *Bryophyllum* population (along with the prediction that it is likely to infest citrus) to preventing an incursion of the pest species on citrus in South Africa and which is still not present in Australia.

The situation that arose through the adventitious establishment of the *Bryophyllum* species of *S. aurantii* into Australia demonstrates the importance of investigating the limits of species gene pools from several research angles (quantified field sampling, host use in the laboratory tests, mating behaviour, phylogeny and population genetics), as some avenues may lead to equivocal results (Walter 2003). When the insects are sampled in an ecologically meaningful way and the results from alternate lines of inquiry are assessed, an accurate delineation of the species limits of the various populations that are involved is more likely than if a single research angle is taken. Our sampling strategy was specifically designed to address the species status of *S. aurantii*, to assess what the quarantine risk to Australia is. It is clear, however, from our results that further sampling across its geographical range in South Africa would yield greater insight into the population structure in its native range and the host plant relationships of each cryptic species. Additional sampling should be designed to test further the species status of other host-associated thrips populations within the *S. aurantii* species complex, with those on mango being a priority. This should be done in conjunction with host testing and behavioural studies of mate recognition to clearly delineate species boundaries within this complex.

Although the host-associated species detected in this study are morphologically identical (Morris and Mound 2004), their associations with their respective hosts are relatively old, having evidently speciated about 1 million years ago (the *Bryophyllum-*associated species) and 4–8 million years ago (the *Gloriosa* associated species). A crucial role for evolutionary biology is to assess the extent of speciation associated with host-associated populations of herbivorous insects that are considered to be generalists. More general information of this nature would be valuable to quarantine, agriculture and biological control (Van Klinken and Edwards 2002). Our study demonstrates the value of an evolutionary approach to applied fields such as quarantine risk assessment and biological control.

## Data archiving statement

Data for this study are available on Dryad: doi:10.5061/dryad.j1b7 h and GenBank (see main text and Table 2 for accession numbers).

## Supporting Information

Figure S1 Delta K plot of multiple runs of the program STRUCTURE across 11 populations of *Scirtothrips aurantii*.

Table S1 Characteristics of eight microsatellite loci developed for *Scirtothrips aurantii*.

Table S2 Summary of genetic data per population and locus; number of individuals scored (N) number of alleles (Na), observed (Ho) and expected (He) heterozygosities, P values for deviation from HW [significance level Bonferroni corrected for multiple comparisons (0.05/88) = 0.00057].

Table S3 Additional primer options for a further 1001 microsatellites identified using the QDD algorithm and results of screening for 24 microsatellite loci tested across host-associated populations of *Scirtothrips auantii* collected in both Australia and South Africa.
