# Supplementary figures and images for: Species limits, quarantine risk and the intrigue of a polyphagous invasive pest with highly restricted host relationships in its area of invasion
Source: Evol Appl. 2013 Aug 21;6(8):1195–207. doi: 10.1111/eva.12096 (PMC3901549; doi:10.1111/eva.12096)

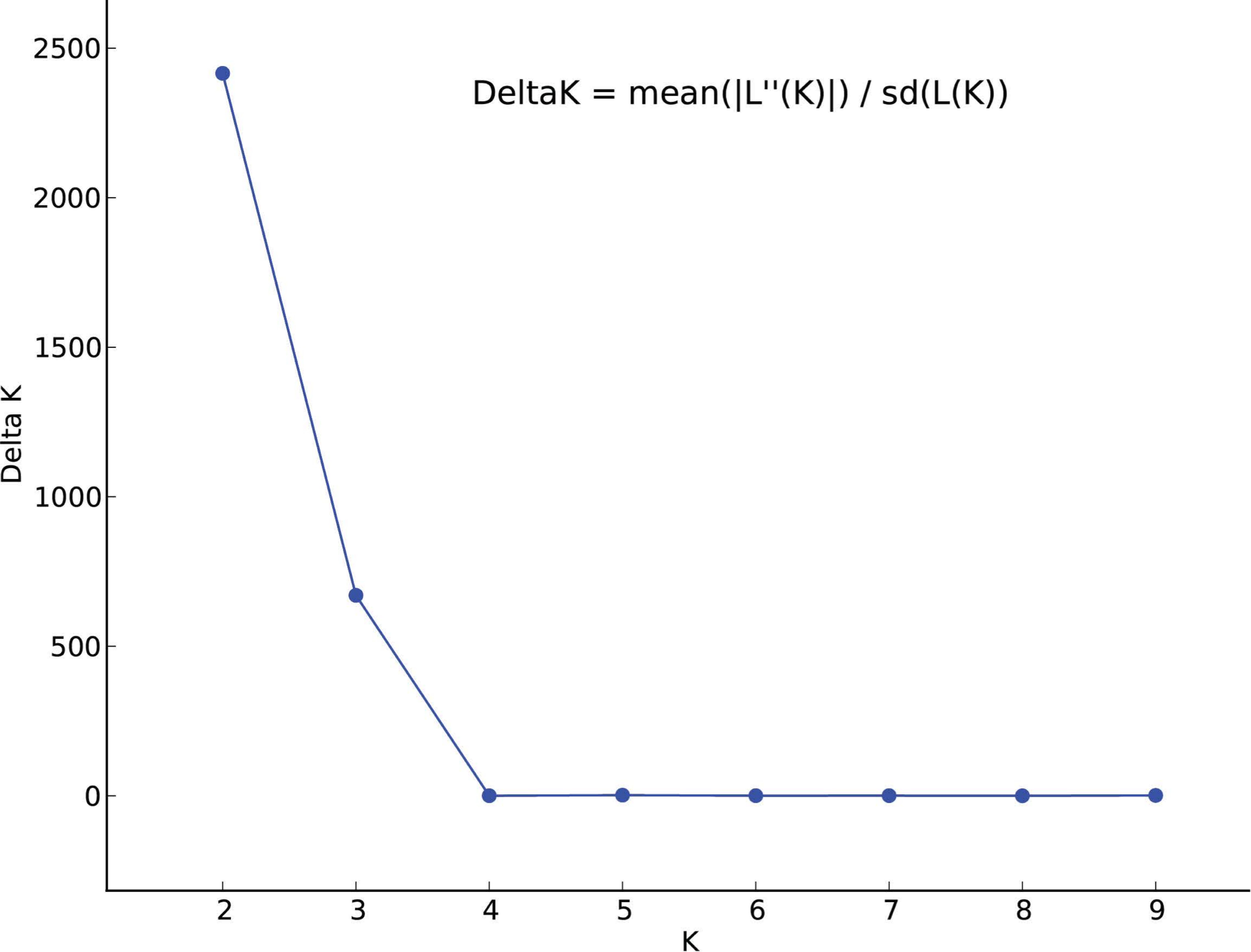

Supplement: Figure S1 — Delta K plot of multiple runs of the program STRUCTURE across 11 populations of Scirtothrips aurantii. [file eva0006-1195-sd1.pdf]
