# Supplementary material for: Species limits, quarantine risk and the intrigue of a polyphagous invasive pest with highly restricted host relationships in its area of invasion
Source: Evol Appl. 2013 Aug 21;6(8):1195–207. doi: 10.1111/eva.12096 (PMC3901549; doi:10.1111/eva.12096)
Supplement: Table S1 — Characteristics of eight microsatellite loci developed for Scirtothrips aurantii. [file eva0006-1195-sd2.doc]

**Table S1: Characteristics of 8 microsatellite loci developed for the thrips *Scirtothrips aurantii*, including the fragment size (bp) and number of alleles (Na) calculated from all populations involved in the microsatellite analysis (see results). Observed (HO) and expected heterozygosities (HE) were calculated in GENALEX 6 (Peakall and Smouse 2006) from 25 screened individuals collected from *Bryophyllum delagoense* from Nelspruit. Conformance to Hardy–Weinberg equilibrium was tested using exact tests implemented in Genepop v. 4.1 (Rousset, 2008). Null allele frequencies (null) were estimated using the expectation maximization algorithm of Dempster et al. (1977) as implemented in FreeNA (Chapuis and Estoup 2007).**

| **Locus** | **Primer sequence (5’-3’)** | **Repeat motif.** | **Fragment size (bp)** | **Na** | **H_o_** | **H_e_** | **PHW** | **Null** |
| --- | --- | --- | --- | --- | --- | --- | --- | --- |
| SACT02 | |  |  |  |  |  |  |  |
|  | F: CGCCCTCTCGATCACCAG | (CCGGG)_6_ | 176-247 | 9 | 0.261 | 0.360 | 0.007 | 0.076 |
|  | R: AGAGCATCCCTACACTGCG |  |  |  |  |  |  |  |
| SACT05 | |  |  |  |  |  |  |  |
|  | F: AGGGTCGCATTAGCACGAG | (CCCG)_6_ | 207-228 | 9 | 0.733 | 0.732 | 0.004 | 0.036 |
|  | R: AAGAGTTTGGTCGATGGCG |  |  |  |  |  |  |  |
| SACT06 | |  |  |  |  |  |  |  |
|  | F: CGGCATTTCTAAAGGCTAAGGG | (CT)_9_ | 142-195 | 6 | 0.537 | 0.700 | 0.001 | 0.120 |
|  | R: GGGCCGTCCGGTTTGAC |  |  |  |  |  |  |  |
| SACT13 | |  |  |  |  |  |  |  |
|  | F: CAGTGCCAATCATCGGTCG | (AGGCC)_11_ | 210-296 | 7 | 0.370 | 0.649 | 0.000 | 0.149 |
|  | R: CACCGTGGCAAATACAGCG |  |  |  |  |  |  |  |
| SACT17 | |  |  |  |  |  |  |  |
|  | F:CCGGACTAGATTCGGACCC | (ACGGG)_8_ | 170-231 | 7 | 0.500 | 0.568 | 0.115 | 0.030 |
|  | R: GGGAGGATGAACCCGATCC |  |  |  |  |  |  |  |
| SACT18 | |  |  |  |  |  |  |  |
|  | F: ACGACGTCAAGTAGGGCTG | (GGGCT)_7_ | 120-166 | 5 | 0.333 | 0.653 | 0.000 | 0.202 |
|  | R: GATCGAAGCTCGGAAACCC |  |  |  |  |  |  |  |
| SACT19 | |  |  |  |  |  |  |  |
|  | F: AACGCAGCATCCTTTGCTC | (CCGGG)_7_ | 113-164 | 7 | 0.349 | 0.432 | 0.861 | 0.089 |
|  | R: TTGTGTTCGCCTTTCGGAC |  |  |  |  |  |  |  |
| SACT52 | |  |  |  |  |  |  |  |
|  | F: GCAAGAAATACAATTCGGCG | (AGT)_15_ | 289-291 | 6 | 0.913 | 0.717 | 0.000 | 0.020 |
|  | R: CAGCATTTGTCAACATTCCAA |  |  |  |  |  |  |  |
